# Coupled enzymatic hydrolysis and ethanol fermentation: ionic liquid pretreatment for enhanced yields

**DOI:** 10.1186/s13068-015-0310-3

**Published:** 2015-09-04

**Authors:** Venkata Prabhakar Soudham, Dilip Govind Raut, Ikenna Anugwom, Tomas Brandberg, Christer Larsson, Jyri-Pekka Mikkola

**Affiliations:** Department of Chemistry, Technical Chemistry and Sustainable Chemical Technology, Chemical-Biological Centre, Umeå University, 901 87 Umeå, Sweden; Department of Chemical and Biological Engineering, Chalmers University of Technology, Kemivägen 10, 412 96 Göteborg, Sweden; Laboratory of Industrial Chemistry and Reaction Engineering, Johan Gadolin Process Chemistry Centre, Åbo Akademi University, 20500 Åbo-Turku, Finland

**Keywords:** Lignocellulose, (Switchable) Ionic liquids, Pretreatment, Hydrolysis, Fermentation, Ethanol, Bio-fuels

## Abstract

**Background:**

Pretreatment is a vital step upon biochemical conversion of lignocellulose materials into biofuels. An acid catalyzed thermochemical treatment is the most commonly employed method for this purpose. Alternatively, ionic liquids (ILs), a class of neoteric solvents, provide unique opportunities as solvents for the pretreatment of a wide range of lignocellulose materials. In the present study, four ionic liquid solvents (ILs), two switchable ILs (SILs) DBU–MEA–SO_2_ and DBU–MEA–CO_2_, as well as two ‘classical’ ILs [Amim][HCO_2_] and [AMMorp][OAc], were applied in the pretreatment of five different lignocellulosic materials: Spruce (*Picea abies*) wood, Pine (*Pinus sylvestris*) stem wood, Birch (*Betula pendula*) wood, Reed canary grass (RCG, *Phalaris arundinacea*), and Pine bark. Pure cellulosic substrate, Avicel, was also included in the study. The investigations were carried out in comparison to acid pretreatments. The efficiency of different pretreatments was then evaluated in terms of sugar release and ethanol fermentation.

**Results:**

Excellent glucan-to-glucose conversion levels (between 75 and 97 %, depending on the biomass and pretreatment process applied) were obtained after the enzymatic hydrolysis of IL-treated substrates. This corresponded between 13 and 77 % for the combined acid treatment and enzymatic hydrolysis. With the exception of 77 % for pine bark, the glucan conversions for the non-treated lignocelluloses were much lower. Upon enzymatic hydrolysis of IL-treated lignocelluloses, a maximum of 92 % hemicelluloses were also released. As expected, the ethanol production upon fermentation of hydrolysates reflected their sugar concentrations, respectively.

**Conclusions:**

Utilization of various ILs as pretreatment solvents for different lignocelluloses was explored. SIL DBU–MEA–SO_2_ was found to be superior solvent for the pretreatment of lignocelluloses, especially in case of softwood substrates (i.e., spruce and pine). In case of birch and RCG, the hydrolysis efficiency of the SIL DBU–MEA–CO_2_ was similar or even better than that of DBU–MEA–SO_2_. Further, the IL [AMMorp][OAc] was found as comparably efficient as DBU–MEA–CO_2._ Pine bark was highly amorphous and none of the pretreatments applied resulted in clear benefits to improve the product yields.

**Electronic supplementary material:**

The online version of this article (doi:10.1186/s13068-015-0310-3) contains supplementary material, which is available to authorized users.

## Background

Second-generation biorefineries based on the exploitation of lignocellulose as the main carbon source, have the potential to produce a variety of products, including bio-fuels, value-added chemicals, materials, heat and electricity [1–3]. However, laboratory scale experiments often report limited product yields due to the complex structure and high crystallinity of the feedstock. As known, lignocelluloses are mainly composed of cellulose, hemicelluloses, and lignin. Cellulose and hemicelluloses are carbohydrate polysaccharides while lignin is a complex aromatic polymer [4]. In combination, these three main components form a complex structure of vegetal biomass. In a typical biomass conversion process, the raw material is pre-treated to improve the accessibility of polysaccharides for their further conversion into monosaccharides. This is typically performed via processing of biomass in environmentally harmful chemicals, such as sulfuric acid that facilitates the hydrolysis and extraction of sugars leaving most of the lignin in the solid residue. Alternatively, lignin can be removed by the use of alkaline solutions or organic solvents, leaving solids rich in sugar polysaccharides. Already, a number of pretreatment methods based on the use of different solvents, e.g., acids, alkali, organic solvents and/or other techniques like steam explosion, ammonia fiber explosion, etc. have been introduced for lignocellulose disruption and are well reviewed [5–11]. The lignin-rich residues obtained from an acid pretreatment can be used as low-value boiler fuel to produce heat and electricity [12]. Moreover, lignin can also be considered as a valuable source of carbon and if selectively removed and recovered it can be used to produce high value derivatives ([5, 6, 13]). After completed pretreatment, enzymes can be used to further degrade and hydrolyze the polysaccharides into monosaccharides which can then be used to produce various products such as alcoholic fuels (e.g., ethanol, butanol) via fermentation [14–17].

If the applied pretreatment is inefficient, the downstream hydrolysis and fermentation are likely to give low product yields [12]. Pretreatment is, therefore, a very important step in lignocellulose conversion processes. Thus, the refinement of lignocellulose pretreatment technologies is necessary to further facilitate enzymatic degradation of polysaccharides, improve product yields, and move closer to an economically viable lignocellulose biorefinery.

Ionic liquids (ILs), salts composed of organic cations and either organic or inorganic anions [18]; these neoteric solvents have lately attracted significant attention due to their ability to dissolve a wide range of organic and inorganic compounds, including lignocellulosic materials [19, 20]. Because of their unique physicochemical properties and potential for associated environmental benefits, ILs are considered to be of interest as potential alternatives to the traditional lignocellulose pretreatment solvents and a variety of ILs have been applied in fractionation and dissolution different lignocelluloses [6, 8, 21–23]. Nevertheless, many ILs are expensive [24] and biomass treatment was in many cases performed at rather low temperatures and with retention times of up to several days [6]. Thus, design of low-cost ILs [24] that efficiently work at high temperatures and with a short processing time is of interest. Among the investigated ones, the use of inexpensive acidic ILs that can be produced on bulk scale is potentially a sustainable approach of lignocellulosic biomass conversion without addition of catalyst [25, 26].

The new acidic switchable ILs (SILs) DBU–MEA–SO_2_ (DBU: 1,8-diazabicyclo[5.4.0]undec-7-ene; MEA: monoethanolamine) and DBU–MEA–CO_2_ have been reported to be efficient for optimal fractionation and selective removal of almost all lignin from the Nordic woody biomass [27, 28]. In addition, among the more commonly applied cellulose-dissolving ILs (CILs) such as [C_2_mim][OAc] [29–32] and [C_4_mim]Cl [29, 30], [Amim][HCO_2_] and [AMMorp][OAc] were proven to be efficient for the dissolution of lignocellulose substrates [33, 34]. Hence, the present study focuses on investigation of above mentioned four (S)ILs in pretreatment, at high temperatures and with a short processing time.

## Results

### Chemical composition of different lignocelluloses

The composition of structural carbohydrates, lignin and extractives of different lignocelluloses used in this study are presented in Table 1. The values in Table 1 are comparable to those reported in the literature [7, 35–37]. Softwood substrates, i.e., spruce and pine, are rich in glucomannans, while both birch (a hard wood substrate) and reed canary grass are rich in glucoxylanes (Table 1). On the contrary, pine bark contains high amounts of arabinoglucans (Table 1). As expected, lignin content of softwood substrates was higher than that of hardwood and reed canary grass. Nevertheless, pine bark displayed the highest lignin content of 40.3 % (dry wt). Pine bark was also exclusively rich in extractives [19.4 % (dry wt)] while the extractives content of other substrates was maximally 4 % (dry wt) (Table 1).

**Table 1:** Relative masses of polysaccharides, lignin, and extractives of different lignocelluloses

Lignocellulose substrate	Component (dry wt%)						
	Arabinan	Galactan	Glucan	Xylan	Mannan	Lignin^a^	Extractives^b^
Spruce wood	1.9 (±0.1)	1.6 (±0.1)	41.0 (±1.1)	4.7 (±0.3)	9.7 (±0.3)	28.3 (±0.1)	2.8 (±0.5)
Pine stem wood	2.0 (±0.1)	2.7 (±0.2)	40.3 (±0.7)	5.2 (±0.4)	9.2 (±0.4)	27.5 (±0.5)	4.0 (±0.4)
Birch wood	0.9 (±0.1)	0.6 (±0.1)	38.0 (±0.6)	17.5 (±0.6)	1.6 (±0.2)	23.7 (±0.6)	3.3 (±0.1)
Reed canary grass	2.6 (<0.1)	1.2 (±0.1)	36.8 (±1.3)	16.4 (±0.5)	0.8 (<0.1)	23.1 (±0.9)	3.5 (±0.7)
Pine bark	12.2 (±0.1)	3.0 (± 0.1)	16.7 (<0.1)	1.7 (±0.2)	1.5 (±0.2)	40.3 (±2.2)	19.4 (±2.1)

Data shown as percentage in dry weight; values represent the mean of three replicates; standard error is given in parentheses

^a^Total lignin, i.e., Klason lignin plus acid soluble lignin

^b^Ethanol extractives

### Enzymatic hydrolysis of (S)IL-treated and non-treated Avicel cellulose

**Fig. 1:**
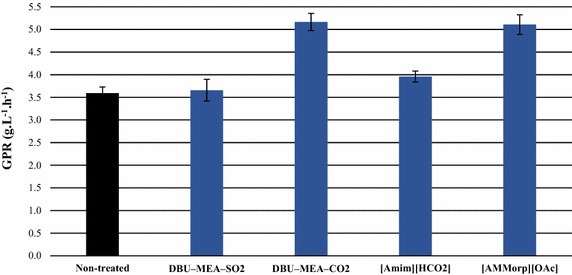
Glucose production rates (GPRs) during 4 h enzymatic hydrolysis of Avicel cellulose. Hydrolysis experiments were performed with either non-treated or (S)IL (SF 2.5)-treated Avicel cellulose

In Initial experiments, a model crystalline cellulose Avicel was treated with various IL solvents at a severity factor (SF) of 2.5 and subsequently subjected to enzymatic hydrolysis. As control, Avicel was hydrolyzed without any pretreatment. After 48 h of enzymatic hydrolysis, the glucose yields (g glucose released/g maximum available glucose) were 0.68, 0.69, 0.81, 0.70, and 0.80 for the non-treated and the samples treated with DBU–MEA–SO_2_, DBU–MEA–CO_2_, [Amim][HCO_2_] and [AMMorp][OAc], respectively. Compared to non-treated material, the glucose production rates (GPRs, calculated from the first 4 h of hydrolysis) were 44, 10 and 42 % higher for the Avicel treated with DBU–MEA–CO_2,_ [Amim][HCO_2_] and [AMMorp][OAc], respectively (Fig. 1). Hence, use of DBU–MEA–CO_2_ and [AMMorp][OAc] resulted in most successful treatments for Avicel cellulose, whereas treatment with DBU–MEA–SO_2_ did not result in any improvement in subsequent enzymatic hydrolysis.

### Enzymatic hydrolysis of non-treated, acid pre-hydrolyzed, and IL-treated lignocellulose substrates

#### Soft wood substrates

**Fig. 2:**
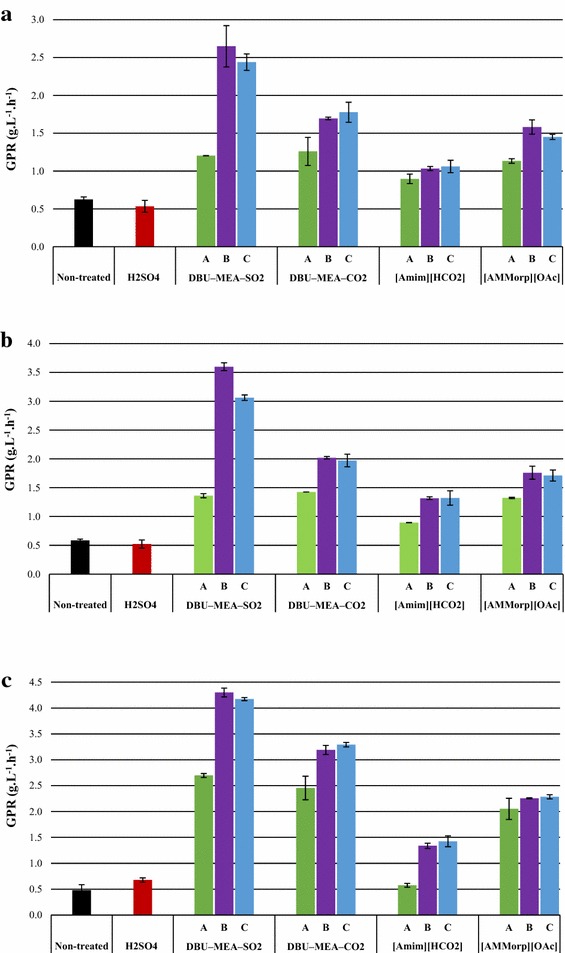
Glucose production rates (GPRs) during 4-h enzymatic hydrolysis of lignocellulose substrates. **a** Spruce wood; **b** Pine stem wood; **c** Birch wood. Enzymatic hydrolysis experiments were performed with either non-treated or H_2_SO_4_-treated (SF 4.1), or (S)IL-treated lignocelluloses. (S)IL treatments were performed at (*A*) SF 2.5 (*B*) SF 3.7 and (*C*) SF 4.1

In case of softwood substrates such as spruce wood and pine stem wood, an acid pre-hydrolysis was not beneficial for the subsequent enzymatic degradation (Figs. 2a, b, 4a, b). The GPRs (~0.6 g L^−1^ h^−1^) and the glucose yields (11–13 %) were similar for the enzymatic hydrolysis of non-treated and the solids of acid pre-hydrolysis (S-APH).

**Fig. 3:**
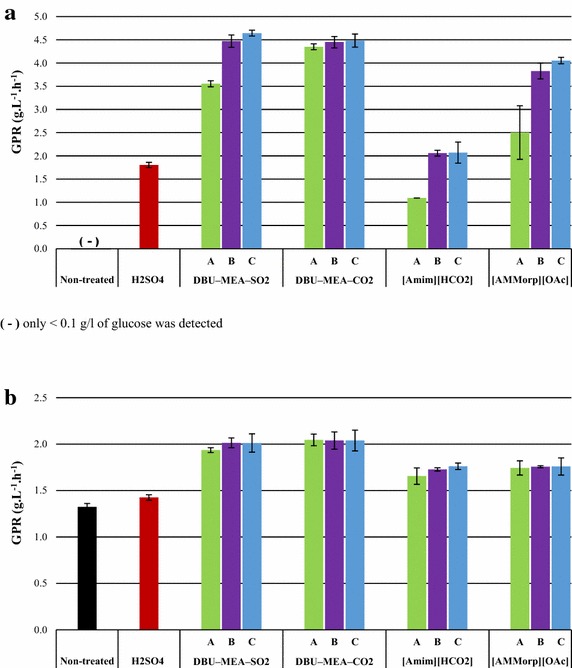
Glucose production rates (GPRs) during 4-h enzymatic hydrolysis of lignocellulose substrates. **a** Reed canary grass; and **b** Pine bark. Enzymatic hydrolysis experiments were performed with either non-treated or H_2_SO_4_-treated (SF 4.1), or (S)IL-treated lignocelluloses. (S)IL treatments were performed at (*A*) SF 2.5 (*B*) SF 3.7 and (*C*) SF 4.1

**Fig. 4:**
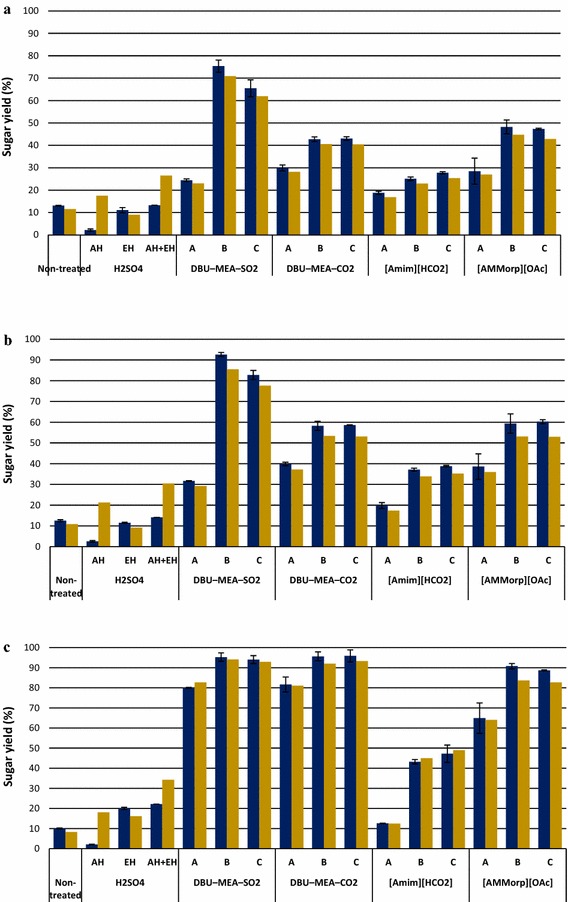
Sugar yield (g sugars released/g available sugars) obtained from the hydrolysis of lignocellulose materials. *Blue bars* represent glucose and *yellow bars* represent total reducing sugars. **a** Spruce wood; **b** Pine stem wood; **c** Birch wood. Enzymatic hydrolysis experiments were performed with either non-treated or H_2_SO_4_-treated (SF 4.1), or (S)IL-treated lignocelluloses. (S)IL treatments were performed at (*A*) SF 2.5 (*B*) SF 3.7 and (*C*) SF 4.1. AH is acid hydrolysate, i.e., liquid fraction obtained from the acid pre-hydrolysis and EH is enzymatic hydrolysate of acid-treated solids

**Fig. 5:**
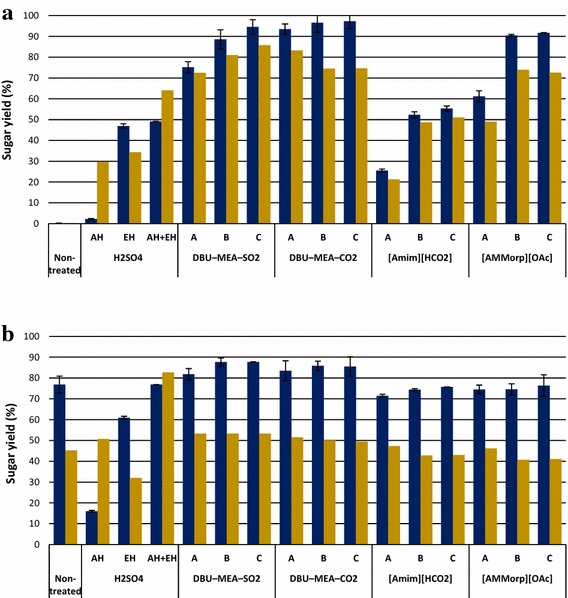
Sugar yield (g sugars released/g available sugars) obtained from the hydrolysis of lignocellulose materials. *Blue bars* represent glucose and *yellow bars* represent total reducing sugars. **a** Reed canary grass; and **b** Pine bark. Enzymatic hydrolysis experiments were performed with either non-treated or H_2_SO_4_-treated (SF 4.1), or (S)IL-treated lignocelluloses. (S)IL treatments were performed at (*A*) SF 2.5 (*B*) SF 3.7 and (*C*) SF 4.1. AH is acid hydrolysate, i.e., liquid fraction obtained from the acid pre-hydrolysis and EH is enzymatic hydrolysate of acid-treated solids

Compared with both non-treated and acid pre-hydrolyzed, softwood substrates originating from IL treatments were readily degraded by enzymes—hydrolysis rates were significantly enhanced and high sugar conversions were observed (Figs. 2a, b, 4a, b). Upon enzymatic hydrolysis of (S)IL-treated substrates, the GPRs and glucose yields were enhanced to a maximum of 1.1–2.7 g L^−1^ h^−1^ and 28–75 % for spruce wood, and 1.3–3.6 g L^−1^ h^−1^ and 39–93 % for pine stem wood. In addition, the hemicellulose recovery 61 and 71 % from the enzymatic hydrolysis of (S)IL spruce and pine, respectively, were slightly higher than that of 57 and 65 % recovered from the combined acid pre-hydrolysis and enzymatic hydrolysis.

DBU–MEA–SO_2_ was the best solvent for the pretreatment of softwood substrates, followed by DBU–MEA–CO_2_ and [AMMorp][OAc]. It should be noted, that IL [AMMorp][OAc] appeared to be in the same magnitude of order as DBU–MEA–CO_2._

### Hardwood substrate

Acid pre-hydrolysis of hard wood such as birch was beneficial and improved the subsequent enzymatic hydrolysis (Figs. 2c, 4c). The GPR increased from 0.5 to 0.7 g L^−1^ h^−1^ and the glucose yields from 10 to 20 % in S-APH samples compared to non-treated birch wood.

Similar to softwoods, enzymatic hydrolysis of (S)IL-treated birch wood was highly efficient. A maximum GPR of 1.4–4.3 g L^−1^ h^−1^ and glucose yields of 47–96 % were obtained from the enzymatic hydrolysis of (S)IL-treated birch wood (Figs. 2c, 4c). In fact, the hemicellulose recovery max 92 % from the enzymatic hydrolysis of (S)IL-treated birch wood was comparably higher than the 56 % recovered from the combined acid pre-hydrolysis and enzymatic hydrolysis of birch wood. The tendency of (S)ILs efficiency for birch wood was similar to that of softwood substrates, but the sugar yields (glucose 95–96 %, overall 93–94 %) were similar irrespective of whether they were DBU–MEA–SO_2_ or DBU–MEA-CO_2_ (Fig. 4c). However, the GPR maximum 3.3 g L^−1^ h^−1^ for the DBU–MEA–CO_2_-treated birch wood was lower than the 4.3 g L^−1^ h^−1^ of DBU–MEA–SO_2_-treated substrate. The glucose yields for the [AMMorp][OAc]-treated birch wood also reached as high as 91 %, but the maximum GPR was only 2.3 g L^−1^ h^−1^.

### Agricultural residues

Surprisingly, unlike any investigated lignocellulose substrates, no sugars were released from the enzymatic hydrolysis of non-treated reed canary grass (Figs. 3a, 5a). However, acid pre-hydrolysis of reed canary grass significantly improved its subsequent enzymatic degradation. Enzymatic hydrolysis of S-APH of reed canary grass resulted in 47 % glucose yield with a GPR of 1.8 g L^−1^ h^−1^.

The hydrolysis efficiency of reed canary grass was further enhanced by (S)IL treatments. Upon enzymatic hydrolysis of (S)IL-treated reed canary grass, a maximum GPRs of 2.1–4.6 g L^−1^ h^−1^ and glucose yields of 55–97 % were obtained. For reed canary grass, both SILs DBU–MEA–SO_2_ and DBU–MEA–CO_2_ were similarly efficient in terms of GPRs and yields. Even though, IL [AMMorp][OAc] was as efficient as SILs still the GPRs for [AMMorp][OAc] treated reed canary grass were slightly lower than for the S-ILs-treated substrates. However, at less sever treatment conditions (i.e. SF 2.5), DBU–MEA–CO_2_ was better solvent than other (S)ILs, resulting a 4.3 g L^−1^ h^−1^ GPR and 94 % glucose yield. Nonetheless, the hemicellulose recovery from the (S)ILs-treated reed canary grass 71 % was lower than the 90 % of recovered from the combined acid pre-hydrolysis and enzymatic hydrolysis.

### Pine bark

Enzymatic hydrolysis of non-treated pine bark was readily degraded, as smoothly as samples pre-treated with either acid or any ILs, by cellulase enzymes (Figs. 3b, 5b) giving a GPR of 1.5 g L^−1^ h^−1^ and glucose yield of 77 %. Compare to non-treated, acid pre-hydrolysis or (S)IL treatment of pine bark had no or only minimal effect on its subsequent enzymatic hydrolysis. Treatment with S-ILs slightly beneficial and improved GPRs max. 2.2 g L^−1^ h^−1^ and glucose yield max. 88 % g L^−1^ glucose.

However, the hemicellulose recovery 88 % and overall sugar yield 83 % obtained from the acid pre-hydrolysis were significantly higher than obtained from either non-treated (17 or 45 %) or (S)IL-treated (23 and 53 %).

### Separate hydrolysis and fermentation of different lignocelluloses after treatment with either sulfuric acid or a SIL DBU–MEA–SO_2_

**Fig. 6:**
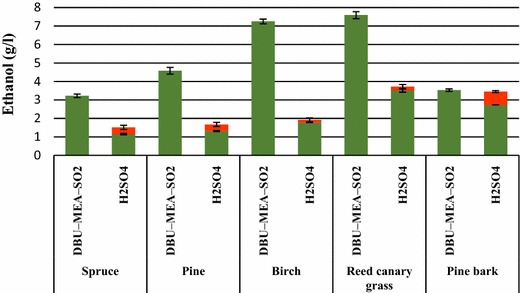
Ethanol produced from the fermentations of lignocellulose hydrolysates. *Green bars* represent ethanol produced from the enzymatic hydrolysates and *red bars* represent ethanol produced from the liquid fraction of acid pre-hydrolysis. Enzymatic hydrolysis experiments were performed with lignocellulose that were first treated with either H_2_SO_4_ (SF 4.1) or with the SIL DBU–MEA–SO_2_ (SF 4.1)

Hydrolysates obtained from the enzymatic hydrolysis of DBU–MEA–SO_2_ treated or acid pre-hydrolyzed substrates were readily fermented to ethanol. Glucose present in the hydrolysates was completely consumed and converted to ethanol within first 10 h of fermentations (Fig. 6). Ethanol concentrations of 1.2, 1.3, 1.8, 3.5, and 2.7 g L^−1^ were obtained from the fermentation of enzymatic hydrolysates of acid pre-hydrolyzed spruce wood, pine stem wood, birch wood, reed canary grass, and pine bark, respectively. The corresponding values for the DBU–MEA–SO_2_ treated substrates were 3.2, 4.6, 7.2, 7.6, and 3.5 g L^−1^, respectively. Even though the overall sugar production was higher for the combined acid and enzymatic hydrolyzed pine bark (Fig. 5b), still the overall ethanol production 3.4 g L^−1^ (0.7 g L^−1^ from acid hydrolysates and 2.7 g L^−1^ from enzymatic hydrolysates) did not exceed that obtained from the hydrolysates of IL-treated substrate (Fig. 6). Evidently, hemicellulose sugars of acid pre-hydrolysates were not consumed by the microorganism *S. cerevisiae* and requires an engineered strain that could use not only glucose but also other lignocellulose derived sugars.

## Discussion

### Non-treated and acid pre-hydrolyzed substrates

The acid pre-hydrolysis procedure, used in our study is known to produce enzymatically digestible biomass, did not benefit the subsequent enzymatic hydrolysis of especially softwood substrates. This observation is, however, consistent with observations reported by Ungurean et al. [2]. Compared to non-treated, less sugars were released from the enzymatic hydrolysis of acid pre-hydrolyzed fir wood [2]. However, the main role of dilute acid pretreatment is to solubilize hemicellulose from the biomass and to make cellulose more accessible for cellulases [2] which is also evident from our study (see Additional file 1: Tables S1–S5). The resistance of acid pre-hydrolyzed material to the hydrolytic enzymes was probably due to the changes in substrate crystallinity and increased enzyme binding capacity of lignin—the major feature that affects enzymatic degradation process [38].

Li et al. [39] investigated the efficiency of dilute acid treatment of lignocellulose substrate. Results indicated that both non-treated and dilute acid-treated samples display no changes in cellulose structure. Also, significant amount of lignin remained in the acid-treated material. Upon enzymatic hydrolysis, cellulases tend to bind on the lignin-rich surfaces—lignin can irreversibly adsorb cellulases [40] causing loss of cellulose degradation. In addition, acid treatment of spruce wood altered the lignin structure leading to increased enzyme adsorption [41, 42]. Moreover, after acid treatment, lignin or lignin carbohydrate complexes may condense on the surface of cellulose fibers [43], thus rendering the fibers less accessible to enzymes.

However, the positive effect of acid pretreatments of birch wood and canary grass could be attributed to their lignin content which contained less lignin than the softwood substrates (Table 1). However, the inhibition of enzymes lignin did not comply for pine bark. Although pine bark contained high amounts of lignin (40.3 % dry wt.), the non-treated substrates were readily degraded by cellulase enzymes.

### Effect of IL treatments

Anugwom et al. [27, 28] investigated the efficiency of SILs MEA–DBU–SO_2_ and MEA–DBU–CO_2_ for the fractionation of woody biomass (i.e. spruce and birch) and reported that both these solvents could remove lignin and produce glucan enriched pulps. However, SILs MEA–DBU–SO_2_ was a better solvent than MEA–DBU–CO_2,_ since it was capable of removing more than 90 % of lignin present in the native substrates whereas MEA–DBU–CO_2_ could remove only up to 50 % [28]. Furthermore, regeneration of substrates via addition of water as anti-solvent (which is performed in our study) could reject the ILs soluble lignin in the solution [44]. Thus, creating a large cellulose accessible surface area for the subsequent enzymatic degradation with no lignin related enzyme inhibition. The improved enzymatic degradation of MEA–DBU–SO_2_ was more likely due to its capacity in selectively removing high amounts of lignin rather than its effect on cellulose crystallinity. In case of MEA–DBU–CO_2_, the improved enzymatic hydrolysis is believed to be due to its synergistic effects. MEA–DBU–CO_2_ is not only capable of removing lignin but also could reduce cellulose crystallinity as evident from the experiments with Avicel cellulose. However, MEA–DBU–CO_2_ treatment of soft wood substrates was less effective probably due to its low lignin removing capacity.

Unlike SILs, [AMMorp][OAc] does not remove lignin. Thus, obviously, the GPRs for the [AMMorp][OAc] treated substrates were lower than the SILs-treated substrates (Figs. 2, 3). However, lignin recovery from [AMMorp][OAc]-treated substrates is rather simple whereas it would require additional efforts in case of SILs. The effect of IL [Amim][HCO_2_] treatments were significantly lower than the any investigated (S)ILs. This is because [Amim][HCO_2_] was less efficient in dissolving cellulose and it does not remove any lignin.

Nevertheless, despite the potential, recovery and reuse of ILs are important to make the process economically feasible. ILs are still more expensive than the conventional pretreatment solvents [6]. The recycling of ILs up to 10–20 times was claimed to allow for process costs per cycle comparable to conventional solvents, hence making ILs as cheaper alternatives as reusable solvents [45]. ILs are comparatively easy to recycle by simply removing the anti-solvent using techniques such as evaporation or distillation [19, 46, 47]. The low-volatile nature of ILs permits distillation of the volatile substances, thus allowing for recovery [48, 49]. It has been already shown that the ILs can be recovered and reused at least up to 5–7 times without decline in their efficiency [22, 49]. However, recovery and reuse of ILs are often a question considering scaled up production of ILs. Nonetheless, ILs that have undergone cellulose regeneration are composed of not only dissolved IL and the anti-solvent but also contain soluble biomass compounds (e.g., lignin, soluble carbohydrates with low molecular weight, degradation products, extractives and others) that were not precipitated in the regeneration step. Recovery of these dissolved compounds is important; for instance, the recovered lignin may potentially serve as a raw material in the production of polymeric materials, and can be tedious.

### Influence of IL treatment conditions on enzymatic hydrolysis

The conditions used for the IL treatments (Table 2) were selected to gain more information about the impact of treatment temperature and residence time on the IL treatments effect on subsequent substrate hydrolysis efficiency.

**Table 2:** Summary of conditions used for the pretreatment of different (lingo)cellulose substrates

Solvent	Temperature and time			
	120 °C (90 min)	160 °C (90 min)	180 °C (60 min)	205 °C (10 min)
DBU–MEA–SO_2_	✓	✓	✓	–
DBU–MEA–CO_2_	✓	✓	✓	–
[Amim][HCO_2_]	✓	✓	✓	–
[AMMorp][OAc]	✓	✓	✓	–
H_2_SO_4_	–	–	–	✓
SF^a^	2.5	3.7	4.1	4.1

^a^Severity factor (SF): SF = log (*t*·exp((*T* − *T*_ref_)/14.75); *t* treatment time in minutes; *T* treatment temperature; *T*_ref_ reference temperature, i.e., 100 °C; 14.75 is an empirically determined constant

At a constant amount, i.e., 5 (w/w) % of biomass loading and upon fixed (S)IL pretreatment time, increasing pretreatment temperature favored and greatly enhanced the enzymatic digestion of lignocellulose substrates similar to observations by Hou et al. [50]. After IL treatment of birch and pine wood, at moderate conditions, the substrates were swollen but not dissolved [51]. It is believed that, at low pretreatment temperatures, the IL molecules mainly swell and disrupt cellulose I lattice, with no appreciable amount of cellulose chains being released into the IL solution. It is also speculated that, at low temperatures, the multilayered structures of plant cell wall and lignin network inhibit dissociation of cellulose chains [50]. However, at higher temperatures, the plant cell walls were destroyed and cellulose chains were released into the IL solution and the highly crystalline cellulose I was transformed into less crystalline cellulose II [52]. Hence, evidently, the highest amount of reducing sugars (and best glucose production rates) was obtained for the substrates treated at 160 and 180 °C (Figs. 2, 3, 4, 5). Shorter (S)IL treatment time (instead of 90 min 60 min) an increase in temperature (from 160 to 180 °C) had no significant effect in terms of subsequent enzymatic hydrolysis. In conclusion, high temperatures and short residence time upon pretreatment of lignocelluloses gave good results.

In general, from our study it was clear that, except or pine bark, lignin is a major barrier and plays an important role in the sugar extraction from lignocellulose substrates. There was a very close correlation between effect of efficiency of pretreatment solvent and lignin content of the lignocellulose. For example for lignin-rich soft wood substrates, lignin-specific SIL DBU–MEA–SO_2_ was the most efficient pretreatment solvent. Nevertheless, in case of the species with low lignin content (hard wood and reed canary grass), DBU–MEA–CO_2_ or [AMMorp][OAc] was the best pretreatment medium_._ Evidently, the differences in the substrate lignin content have an impact on the results of any pretreatment as reported before [53].

## Conclusions

The potential of different (S)ILs as pretreatment solvents upon conversion of several lignocelluloses into bioethanol was investigated. It was demonstrated that (S)IL treatments could significantly improve the enzymatic hydrolysis of biomass. SILs were in relative terms better pretreatment solvents, especially in case of softwood substrates. The SIL DBU–MEA–SO_2_ was the best pretreatment media for woody substrates liberating both glucose and hemicellulose sugars. Nevertheless, in case of Pine bark, the combined acid treatment and enzymatic hydrolysis gave better results than what could be achieved with any (S)IL preprocessing. However, hydrolysates obtained from the enzymatic hydrolysis of (S)IL-treated lignocelluloses were readily fermented to ethanol and the yields were up to four times higher compared to the case when combined acid and enzymatic hydrolysis was employed. Thus, (S)IL-mediated preprocessing of lignocellulosic biomass can offer advantages over conventional acid treatments.

However, even though the (S)ILs investigated in this study were highly efficient, still challenges remain in their applications such as recovery of any (S)IL-degraded species (notably lignin and sugar polysaccharides) and potentially challenging recycling of (S)ILs.

## Methods

### Substrates

A variety of lignocellulose materials, including those of soft wood, hard wood and agriculture residues were targeted in the current study. Norway spruce wood, pine stem wood, birch saw dust, pine bark, and reed canary grass were the species studied. The substrates were first air dried at room temperature until a constant weight and moisture content less than 10 (w/w) %, was achieved. Afterwards, they were milled and sieved to an even particle size <1 mm using a Wiley mill and stored in sealed plastic bags at room temperature until further use. The dry-matter content of the substrates was determined using a Sartorius MA30 Electronic Moisture Analyzer (Germany) through heating by infrared rays and determination of weight loss. Along with native lignocellulose materials, a commercial microcrystalline cellulose substrate Avicel^®^ PH-101 (Sigma-Aldrich) was also used in the investigation for the sake of comparison.

The chemical composition of lignocelluloses in terms of structural carbohydrate content, lignin and extractives were analyzed according to National Renewable Energy Laboratory (NREL) analytical procedures [54, 55].

### Ionic liquids

SILs DBU–MEA–SO_2_ and DBU–MEA–CO_2_ were prepared as described in Anugwom et al. [27, 28]. An equimolar mixture of DBU and MEA was bubbled with either SO_2_ or CO_2_ gas under rigorous stirring and the reactions were performed until the complete formation of SILs. [Amim][HCO_2_] was synthesised as reported earlier in Soudham et al. [33].

The new IL [AMMorp][*OAc*] was prepared as follows: Amberlite IRA-400(R-OH) (10.0 g in deionized water) was loaded in a chromatography column (20 × 1.5 cm) and then 1.0 M sodium acetate solution (100 mL) was passed through the column to facilitate ion exchange. After, the column was thoroughly washed with deionized water until the eluent pH ~7 was obtained. The corresponding bromide precursor, *N*-allyl-*N*-methylmorpholinium bromide (4.44 g in 50 mL deionized water), solution was carefully loaded and passed through the column followed by deionized water (50 mL). The eluent containing [AMMorp][OAc] was collected and water evaporated. Then the IL was dried at 60 °C under high vacuum (4 × 10^−2^ mbar) to remove residual water.

### Pretreatment procedures

Pretreatment of different cellulosic substrates (50 mg) with either various IL solvents (950 mg) or 1 (w/w) % H_2_SO_4_ (950 mg) were performed in 12 mL borosilicate glass tubes with polytetrafluoroethylene (PTFE)-lined screw caps. A pressure reactor (Teflon lined stainless steel, homemade, 500 mL) with silicon oil was preheated to a desired treatment temperature using a furnace (*T*_max_ < 1100 °C) equipped with B 180 controller and NiCr-Ni thermocouple (Nabertherm Muffle furnace, Model No. LVT 9/11, Germany). Glass tubes with cellulosic substrates and ionic liquids were then immersed into the preheated reactor and closed tightly. The reactor was then placed in the furnace and the desired reaction conditions were set (Table 2). After treatment, the reactor was removed from the furnace; the tubes were removed from the reactor and allowed to cool to room temperature. The IL-treated cellulose rich substrates were then precipitated by simply adding 6 g of anti-solvent (in our case deionized water) to the pretreated solutions. Afterwards, the solids were separated by vigorous mixing and centrifugation for 7 min and 3000 rpm (Allegra^®^ 25R centrifuge, BECKMAN COULTER, USA). The IL rich supernatants were decanted and the solids were subsequently washed as described above, using 3 × 6 g anti-solvent and 1 × 6 g 50 mM citrate buffer pH 5.8. These are hereafter referred to as regenerated substrates. In the case of lignocelluloses pre-hydrolyzed with H_2_SO_4,_ the solid and liquid fractions were separated by centrifugation. The collected liquid fractions (acid hydrolysates—AHs) were stored at –80 °C and the solids were washed with deionized water and citrate buffer as mentioned earlier. The obtained regenerated substrates from IL treatments and the solids from acid pre-hydrolysis (S-AH) were then lyophilized (Alpha 2-4 LSC Freeze Dryer, Martin Christ Gefriertrocknungsanlagen GmbH, Germany) to remove any residual liquids, thus avoiding uneven dilutions upon their enzymatic hydrolysis.

To compare the efficiency of different treatments used in this study, the parameter severity factor (SF) was employed, which incorporates the treatment time and temperature (see the equation below). It is generally used to assess various individual lignocellulose pretreatment strategies [56].

$${\text{SF}} = {\text{log }}\left( {t \cdot { \exp }((T - T_{\text{ref}} )/14.75)} \right)$$

In the above equation, *t* is the treatment time in minutes, *T* is the treatment temperature, *T*_ref_ is the reference temperature (i.e., 100 °C) and 14.75 is an empirically determined constant.

### Enzymatic hydrolysis

Enzymatic hydrolysis experiments of non-treated and regenerated cellulosic substrates were carried out in 12 mL glass tubes with 930 mg of 50 mM citrate buffer pH 5.8 and 20 mg of Cellic CTec2, activity 128.6 FPU/g, state-of-the-art enzyme mix (Novozymes). Hydrolysis reactions were performed for 48 h in a shaking incubator (IKA^®^ KS 4000, control IKA^®^-Werke GmbH & Co. KG, Germany) set at 50 °C and 200 rpm. At defined time intervals (4, 24, and 48 h after the addition of enzymes), samples of 50 μL (enzymatic hydrolysates—EHs) were collected from the hydrolysis systems and stored at −80 °C.

### Separate hydrolysis and fermentation

Lignocellulose samples, 0.25 g (dry weight), were pretreated at a severity factor of 4.1 (Table 2) with either the SIL DBU–MEA–SO_2_ (4.75 g) or 1 (w/w) % H_2_SO_4_ (4.75 g) in 10 mL glass tubes. Hydrolysis of regenerated substrates 0.3 g, obtained after the treatments and lyophilization, was performed in the presence of citrate buffer pH 5.8 (5.58 g) and enzyme (0.12 g) mix. Thus, the pretreatments and enzymatic hydrolysis experiments were performed (as described in earlier sections) with an increased overall reaction volume, but in equal concentrations. After enzymatic hydrolysis, the solid and liquid fractions were separated by centrifugation and the sugar-rich liquid fractions were used for ethanol fermentations as described below.

The yeast *S. cerevisiae, Thermosacc* inoculum was prepared in a 2 L cotton-plugged shake flask with 1 L YPD medium (10 g L^−1^ yeast extract, 20 g L^−1^ peptone, 20 g L^−1^d-glucose). The medium was inoculated and incubated with agitation (200 rpm) at 30 °C and the cells were harvested in late exponential growth phase by centrifugation (Hermle Z206A, Hermle Labortechnik GmbH, Wehingen, Germany) at 1500*g* for 5 min. The harvested cells were concentrated and re-suspended in an appropriate amount of sterile water to achieve a cell density of 27 g L^−1^ (dry weight). Fermentations of liquid hydrolysates, obtained from both the acid pre-hydrolysis and enzymatic hydrolysis, were performed in 12 mL screw capped plastic tubes. Sugar-rich liquid hydrolysates (2.3 mL of each) were added to the tubes along with 0.05 mL nutrient solution (75 g L^−1^ yeast extract, 37.5 g L^−1^ (NH_4_)_2_HPO_4_, 1.875 g L^−1^ MgSO_4_·7H_2_O, 119.1 g L^−1^ NaH_2_PO_4_.H_2_O), and 0.15 mL of yeast inoculum. Thus, the fermentation broths contained a total liquid volume of 2.5 mL and had a yeast cell density of 1.6 g L^−1^ dry weight. After inoculation, the tubes were incubated at 30 °C and stirred (at 200 rpm) in an orbital shaking incubator (IKA-Werke) for 24 h. Samples of 100 µL were collected at defined time intervals and stored at −80 °C until further analysis.

### Analysis

Samples collected from different experiments of this study were centrifuged (Centrifuge: Thermo Scientific, Germany) at 21,000*g* for 5 min and the supernatants were used for chemical analysis by either using ion chromatography (IC; ICS 3000, Dionex Corporation, USA) or High-Performance Liquid Chromatography (HPLC; Dionex^TM^ UltiMate 3000, Dionex Corporation, USA).

The monosaccharide (i.e., arabinose, galactose, glucose, mannose, and xylose) concentrations were analyzed in a similar manner as in Wang et al. [57] by Ion Chromatography using a CarboPac PA1 column (Bio-Rad Laboratories). Ethanol concentrations were measured by HPLC equipped with a Rezex ROA-Organic acid H column (containing sulfonated styrene–divinylbenzene spheres in 8 % cross-link forms, 300 × 7.8 mm, Phenomenex^®^, USA) as previously described in Soudham et al. [58].

## Additional file

Additional file 1:
**Tables S1–S5.** Sugars released from the hydrolysis of lignocellulose materials. Sugars released from the enzymatic hydrolysis of lignocelluloses non-treated and treated with either H_2_SO_4_ (205 °C for 10 min) or (S)IL solvents at (A) 120 °C for 90 min (B) 160 °C for 90 min and (C) 180 °C for 60 min. Sugars released from the acid pre-hydrolysis are also shown. AH is acid hydrolysate and EH is enzymatic hydrolysate

## Abbreviations

[Amim][HCO_2_]: 1-allyl-3-methylimidazolium formate
[AMMorp][OAc]: *N*-allyl-*N*-methylmorpholinium acetate
[C_2_mim][OAc]: 1-ethyl-3-methylimidazolium acetate
[C_4_mim]Cl: 1-butyl-3-methylimidazolium chloride
AH: acid hydrolysate
DBU: 1,8-diazabicycloundec-7-ene
EH: enzymatic hydrolysate
GPR: glucose production rate
IL: ionic liquid
MEA: monoethanolamine
RCG: reed canary grass
S-APH: solids obtained from the acid pre-hydrolysis
SF: severity factor
SIL: switchable ionic liquid
