# Supplementary material for: Coupled enzymatic hydrolysis and ethanol fermentation: ionic liquid pretreatment for enhanced yields
Source: Biotechnol Biofuels. 2015 Sep 4;8:135. doi: 10.1186/s13068-015-0310-3 (PMC4558776; doi:10.1186/s13068-015-0310-3)
Supplement: Additional file 1: — Tables S1–S5. Sugars released from the hydrolysis of lignocellulose materials. Sugars released from the enzymatic hydrolysis of lignocelluloses non-treated and treated with either H2SO4 (205 °C for 10 min) or (S)IL solvents at (A) 120 °C for 90 min (B) 160 °C for 90 min and (C) 180 °C for 60 min. Sugars released from the acid pre-hydrolysis are also shown. AH is acid hydrolysate and EH is enzymatic hydrolysate [file 13068_2015_310_MOESM1_ESM.docx]

**Appendix A**

**Table A.1** Spruce wood

| **Pre-treatment solvent** | | **Sugar concentration (g/l)** | | | | | |
| --- | --- | --- | --- | --- | --- | --- | --- |
|  |  | **Arabinose** | **Galactose** | **Glucose** | **Xylose** | **Mannose** | **Total** |
| Non-treated | | 0.1 (<0.01) | 0.1 (<0.01) | 3.0 (0.04) | 0.2 (0.01) | 0.5 (0.01) | 3.8 |
| H_2_SO_4_ | AH | 0.99 (0.01) | 0.61 (0.04) | 0.50 (0.11) | 1.49 (0.10) | 2.14 (0.33) | 5.7 |
|  | EH | <0.1 | <0.1 | 2.5 (0.26) | 0.1 (0.01) | 0.3 (0.02) | 2.9 |
|  | AH+EH | 1.0 | 0.6 | 3.0 | 1.6 | 2.4 | 8.7 |
| DBU–MEA–SO_2_ | A | 0.1  (0.01) | 0.2 (<0.01) | 5.6 (0.12) | 0.5 (0.02) | 1.1 (0.03) | 7.5 |
|  | B | 0.4  (0.01) | 0.2  (0.01) | 17.2 (0.61) | 2.0 (0.11) | 3.5 (0.52) | 23.3 |
|  | C | 0.4  (0.02) | 0.2 (<0.01) | 14.9 (0.86) | 1.7 (0.15) | 3.1 (0.29) | 20.3 |
| DBU–MEA–CO_2_ | A | 0.2  (0.01) | 0.1  (0.02) | 6.8 (0.30) | 0.7 (0.05) | 1.5 (0.06) | 9.2 |
|  | B | 0.2  (0.01) | 0.2  (0.01) | 9.7 (0.23) | 1.0 (0.03) | 2.2 (0.07) | 13.3 |
|  | C | 0.2  (0.01) | 0.2 (<0.01) | 9.8 (0.18) | 1.0 (0.09) | 2.1 (0.14) | 13.3 |
| [Amim](HCO_2_) | A | 0.1  (0.01) | 0.1  (0.02) | 4.3 (0.15) | 0.3 (0.02) | 0.7 (0.04) | 5.5 |
|  | B | 0.1  (<0.01) | 0.1 (<0.01) | 5.7 (0.19) | 0.6 (0.01) | 1.0 (0.04) | 7.5 |
|  | C | 0.1  (<0.01) | 0.1 (<0.01) | 6.3 (0.11) | 0.7 (0.05) | 1.1 (0.08) | 8.3 |
| [AMMorp](OAc) | A | 0.1  (0.04) | 0.1  (0.03) | 6.5 (1.32) | 0.7 (0.24) | 1.4 (0.31) | 8.8 |
|  | B | 0.3  (0.05) | 0.1  (0.01) | 11.0 (0.72) | 1.5 (0.22) | 1.8 (0.23) | 14.7 |
|  | C | 0.2  (<0.01) | 0.1 (<0.01) | 10.8 (0.07) | 1.3 (0.06) | 1.6 (0.03) | 14.0 |

**Table A.2** Pine stem wood

| **Pre-treatment solvent** | | **Sugar concentration (g/l)** | | | | | |
| --- | --- | --- | --- | --- | --- | --- | --- |
|  |  | **Arabinose** | **Galactose** | **Glucose** | **Xylose** | **Mannose** | **Total** |
| Non-treated | | 0.1 (<0.01) | 0.1 (<0.01) | 2.8 (0.11) | 0.2 (0.01) | 0.4 (0.03) | 3.6 |
| H_2_SO_4_ | AH | 1.12 (0.04) | 0.97 (<0.01) | 0.57 (0.08) | 2.01 (<0.01) | 2.38 (0.08) | 7.0 |
|  | EH | <0.1 | <0.1 | 2.6 (0.06) | 0.2 (<0.01) | 0.3 (0.01) | 3.0 |
|  | AH+EH | 1.1 | 1.0 | 3.2 | 2.2 | 2.6 | 10.1 |
| DBU–MEA–SO_2_ | A | 0.2  (<0.01) | 0.1 (<0.01) | 7.1 (0.04) | 0.9 (0.03) | 1.4 (0.03) | 9.7 |
|  | B | 0.5  (0.01) | 0.2 (<0.01) | 20.7 (0.22) | 2.7 (0.04) | 4.1 (0.33) | 28.3 |
|  | C | 0.5  (0.01) | 0.2  (0.01) | 18.5 (0.49) | 2.6 (0.09) | 3.9 (0.09) | 25.7 |
| DBU–MEA–CO_2_ | A | 0.2  (<0.01) | 0.2  (0.01) | 8.9 (0.19) | 1.1 (0.02) | 1.9 (0.04) | 12.3 |
|  | B | 0.3  (0.01) | 0.2 (<0.01) | 13.0 (0.49) | 1.6 (0.07) | 2.6 (0.01) | 17.7 |
|  | C | 0.3  (<0.01) | 0.2 (<0.01) | 13.1 (0.02) | 1.6 (0.05) | 2.4 (0.12) | 17.6 |
| [Amim](HCO_2_) | A | 0.1  (0.01) | 0.1  (0.01) | 4.4 (0.32) | 0.4  (0.04) | 0.7  (0.06) | 5.7 |
|  | B | 0.2  (<0.01) | 0.1 (<0.01) | 8.3 (0.16) | 1.2 (<0.01) | 1.5 (0.03) | 11.2 |
|  | C | 0.2  (<0.01) | 0.1 (<0.01) | 8.7 (0.07) | 1.2 (0.01) | 1.5 (0.03) | 11.7 |
| [AMMorp](OAc) | A | 0.2  (0.04) | 0.1  (0.01) | 8.6 (1.38) | 1.2 (0.29) | 1.7 (0.29) | 11.9 |
|  | B | 0.3  (0.02) | 0.1 (<0.01) | 13.3 (1.04) | 1.9 (0.12) | 2.0 (0.05) | 17.6 |
|  | C | 0.3  (<0.01) | 0.1 (<0.01) | 13.5 (0.21) | 1.8 (0.04) | 1.9 (0.04) | 17.5 |

**Table A.3** Birch wood

| **Pre-treatment solvent** | | **Sugar concentration (g/l)** | | | | | |
| --- | --- | --- | --- | --- | --- | --- | --- |
|  |  | **Arabinose** | **Galactose** | **Glucose** | **Xylose** | **Mannose** | **Total** |
| Non-treated | | 0.1 (<0.01) | 0.1 (<0.01) | 2.1 (0.06) | 0.3 (0.02) | 0.1 (0.01) | 2.7 |
| H_2_SO_4_ | AH | 0.44 (0.01) | 0.31 (0.01) | 0.44  (0.03) | 4.59  (0.01) | 0.14  (0.02) | 5.9 |
|  | EH | 0.0 (<0.01) | 0.0 (<0.01) | 4.2 (0.12) | 0.9 (<0.01) | 0.1 (0.01) | 5.3 |
|  | AH+EH | 0.5 | 0.3 | 4.7 | 5.5 | 0.3 | 11.2 |
| DBU–MEA–SO_2_ | A | 0.1  (<0.01) | 0.1  (0.01) | 16.9 (0.05) | 9.2 (0.01) | 0.8  (<0.01) | 27.1 |
|  | B | 0.1  (<0.01) | 0.1 (<0.01) | 20.1 (0.45) | 9.8 (0.06) | 0.6  (0.02) | 30.8 |
|  | C | 0.1  (<0.01) | 0.1 (<0.01) | 19.8 (0.42) | 9.7 (0.22) | 0.7  (0.04) | 30.5 |
| DBU–MEA–CO_2_ | A | 0.1  (0.00) | 0.1 (<0.01) | 17.2 (0.79) | 8.5 (0.15) | 0.7  (0.04) | 26.6 |
|  | B | 0.1  (<0.01) | 0.1 (<0.01) | 20.2 (0.47) | 9.0 (0.23) | 0.8  (0.02) | 30.2 |
|  | C | 0.1  (<0.01) | 0.1 (<0.01) | 20.2 (0.64) | 9.4 (0.44) | 0.8  (<0.01) | 30.6 |
| [Amim](HCO_2_) | A | 0.1  (<0.01) | 0.1  (0.02) | 2.7  (0.02) | 1.1 (0.02) | 0.1  (0.01) | 4.1 |
|  | B | <0.1  (<0.01) | 0.1 (<0.01) | 9.1  (0.23) | 5.2 (0.05) | 0.3  (0.10) | 14.8 |
|  | C | <0.1  (<0.01) | 0.1 (<0.01) | 10.0 (0.91) | 5.6 (0.35) | 0.4  (0.03) | 16.1 |
| [AMMorp](OAc) | A | <0.1  (0.01) | 0.1 (<0.01) | 13.7 (1.60) | 6.7 (0.60) | 0.4  (0.13) | 21.0 |
|  | B | <0.1  (<0.01) | 0.1 (<0.01) | 19.2 (0.28) | 7.7 (0.07) | 0.4  (<0.01) | 27.4 |
|  | C | <0.1  (<0.01) | 0.1 (<0.01) | 18.7 (0.05) | 7.9 (0.19) | 0.3  (0.12) | 27.1 |

**Table A.4** Reed canary grass

| **Pre-treatment solvent** | | **Sugar concentration (g/l)** | | | | | |
| --- | --- | --- | --- | --- | --- | --- | --- |
|  |  | **Arabinose** | **Galactose** | **Glucose** | **Xylose** | **Mannose** | **Total** |
| Non-treated | | <0.1 | <0.1 | <0.1 | <0.1 | <0.1 | 0.1 |
| H_2_SO_4_ | AH | 1.44 (0.11) | 0.65 (0.07) | 0.46 (0.05) | 6.91 (0.71) | 0.15 (0.02) | 9.6 |
|  | EH | <0.1 | <0.1 | 9.6 (0.22) | 1.3 (<0.01) | 0.2 (0.01) | 11.1 |
|  | AH+EH | 1.4 | 0.7 | 10.0 | 8.2 | 0.3 | 20.7 |
| DBU–MEA–SO_2_ | A | 0.7  (0.09) | 0.1 (<0.01) | 15.4 (0.55) | 6.9 (0.82) | 0.3  (0.01) | 23.4 |
|  | B | 0.8  (0.01) | 0.1 (<0.01) | 18.1 (0.93) | 6.8 (0.38) | 0.3 (0.14) | 26.2 |
|  | C | 0.8  (0.08) | 0.2 (<0.01) | 19.3 (0.72) | 7.1 (0.66) | 0.4 (0.02) | 27.7 |
| DBU–MEA–CO_2_ | A | 0.8  (0.14) | 0.1 (<0.01) | 19.1 (0.51) | 6.6 (0.41) | 0.2 (0.01) | 26.9 |
|  | B | 0.5  (0.09) | 0.1 (<0.01) | 19.7 (0.93) | 3.5 (0.27) | 0.3 (0.01) | 24.1 |
|  | C | 0.5  (0.07) | 0.1 (<0.01) | 19.9 (0.71) | 3.5 (0.49) | 0.2 (0.04) | 24.2 |
| [Amim](HCO_2_) | A | 0.1  (<0.01) | 0.1  (0.01) | 5.2 (0.15) | 1.2 (0.07) | 0.2 (0.03) | 6.9 |
|  | B | 0.3  (0.02) | 0.1 (<0.01) | 10.7 (0.30) | 4.4 (0.15) | 0.2 (0.04) | 15.7 |
|  | C | 0.3  (0.01) | 0.1 (<0.01) | 11.3 (0.24) | 4.6 (0.15) | 0.2 (0.09) | 16.5 |
| [AMMorp](OAc) | A | 0.4  (0.22) | 0.1  (0.01) | 12.5 (0.55) | 2.7 (0.05) | 0.2 (0.05) | 15.9 |
|  | B | 0.6  (<0.01) | 0.1  (0.01) | 18.5 (0.11) | 4.5 (0.16) | 0.3 (0.01) | 23.9 |
|  | C | 0.5  (<0.01) | 0.1  (0.02) | 18.7 (0.03) | 3.9 (0.01) | 0.2 (0.09) | 23.5 |

**Table A.5** Pine bark

| **Pre-treatment solvent** | | **Sugar concentration (g/l)** | | | | | |
| --- | --- | --- | --- | --- | --- | --- | --- |
|  |  | **Arabinose** | **Galactose** | **Glucose** | **Xylose** | **Mannose** | **Total** |
| Non-treated | | 1.0 (0.04) | 0.2  (0.02) | 7.1 (0.38) | 0.3 (0.02) | 0.4 (0.03) | 8.9 |
| H_2_SO_4_ | AH | 6.48 (0.01) | 1.12 (0.02) | 1.48 (0.04) | 0.66 (0.01) | 0.23 (<0.01) | 10.0 |
|  | EH | <0.1 | <0.1 | 5.7 (0.07) | 0.3 (0.01) | 0.3 (0.03) | 6.3 |
|  | AH+EH | 6.5 | 1.1 | 7.1 | 0.9 | 0.6 | 16.3 |
| DBU–MEA–SO_2_ | A | 1.6  (0.07) | 0.3  (0.01) | 7.6 (0.25) | 0.3 (0.01) | 0.7 (0.03) | 10.5 |
|  | B | 0.8  (0.11) | 0.3 (<0.01) | 8.1 (0.18) | 0.5 (0.02) | 0.8 (0.02) | 10.5 |
|  | C | 0.8  (0.09) | 0.3  (0.01) | 8.1 (0.01) | 0.5 (0.01) | 0.8 (<0.01) | 10.5 |
| DBU–MEA–CO_2_ | A | 1.1  (0.12) | 0.3  (0.04) | 7.7 (0.45) | 0.3 (0.03) | 0.7 (0.07) | 10.1 |
|  | B | 0.6  (0.29) | 0.2 (<0.01) | 8.0 (0.21) | 0.3 (0.02) | 0.7 (0.05) | 9.8 |
|  | C | 0.6  (0.03) | 0.2  (0.01) | 7.9 (0.43) | 0.3 (0.01) | 0.6 (0.04) | 9.7 |
| [Amim](HCO_2_) | A | 1.6  (<0.01) | 0.3  (0.01) | 6.6 (0.07) | 0.3 (0.01) | 0.5 (<0.01) | 9.3 |
|  | B | 0.5  (0.02) | 0.3  (0.01) | 6.9 (0.04) | 0.3 (<0.01) | 0.5 (0.01) | 8.4 |
|  | C | 0.4  (0.01) | 0.3  (0.01) | 7.0 (<0.01) | 0.3 (0.01) | 0.4 (0.02) | 8.5 |
| [AMMorp](OAc) | A | 1.3  (0.41) | 0.2  (0.01) | 6.9 (0.19) | 0.2 (0.05) | 0.4 (0.09) | 9.1 |
|  | B | 0.4  (0.02) | 0.2  (0.01) | 6.9 (0.25) | 0.3 (0.04) | 0.3 (0.03) | 8.0 |
|  | C | 0.3  (0.09) | 0.1 (<0.01) | 7.1 (0.48) | 0.3 (0.05) | 0.3 (0.03) | 8.1 |
